# Lipoprotein-Associated Phospholipase A2 Activity Predicts Cardiovascular Events in High Risk Coronary Artery Disease Patients

**DOI:** 10.1371/journal.pone.0048171

**Published:** 2012-10-31

**Authors:** Giuseppe Maiolino, Luigi Pedon, Maurizio Cesari, Anna Chiara Frigo, Robert L. Wolfert, Marlena Barisa, Leopoldo Pagliani, Giacomo Rossitto, Teresa Maria Seccia, Mario Zanchetta, Gian Paolo Rossi

**Affiliations:** 1 Divisione di Cardiologia, Ospedale di Cittadella, Cittadella, Italy; 2 Department of Medicine, Internal Medicine 4, University of Padua, Padua, Italy; 3 Department of Environmental Medicine and Public Health, University of Padua, Padua, Italy; 4 diaDexus, San Francisco, California, United States of America; University Medical Center Utrecht, The Netherlands

## Abstract

**Objective:**

Lipoprotein-associated phospholipase A2 (Lp-PLA2) is deemed to play a role in atherosclerosis and plaque destabilization as demonstrated in animal models and in prospective clinical studies. However, most of the literature is either focused on high-risk, apparently healthy patients, or is based on cross sectional studies. Therefore, we tested the hypothesis that serum Lp-PLA2 mass and activity are useful for predicting cardiovascular (CV) events over the coronary atherosclerotic burden and conventional risk factors in high-risk coronary artery disease patients.

**Methods and Results:**

In a prospective cohort study of 712 Caucasian patients, who underwent coronary angiography and measurement of both Lp-PLA2 mass and activity at baseline, we determined incident CV events at follow-up after splitting the patients into a high and a low Lp-PLA2 mass and activity groups based on ROC analysis and Youden index. Kaplan-Meier and propensity score matching analysis were used to compare CV event-free survival between groups. Follow-up data were obtained in 75% of the cohort after a median of 7.2 years (range 1–12.7 years) during which 129 (25.5%) CV events were observed. The high Lp-PLA2 activity patients showed worse CV event-free survival (66.7% vs. 79.5%, p = 0.023) and acute coronary syndrome-free survival (75.4% vs. 85.6%, p = 0.04) than those in low Lp-PLA2 group.

**Conclusions:**

A high Lp-PLA2 activity implies a worse CV prognosis at long term follow up in high-risk Caucasian patients referred for coronary angiography.

## Introduction

Lipoprotein-associated phospholipase A2 (Lp-PLA2) is a Ca^2+^-independent protein produced by monocytes/macrophages [1], which acts by hydrolyzing the sn-2 acyl chain of the phospholipid substrate [2]. It circulates in plasma associated preferably with the densest and more electronegative fractions of low-density lipoproteins (LDLs) [3], and to a much lesser extent to high-density lipoproteins (HDLs) [4].

It has been contended that Lp-PLA2 can be pro-atherogenic owing to the fact that it was found within atherosclerotic plaques where it co-localized with macrophages and foam cells [5], particularly in regions abundant in lipids and oxidation products, in thin-cap fibroatheromas, necrotic cores of ruptured plaques, and in advanced atherosclerotic lesions rich in apoptotic macrophages [6]. The pro-atherogenic mechanism of Lp-PLA2 could be related to the hydrolysis of oxidized phospholipids on the LDL surface [7], forming oxidized fatty acids and lysophosphatidylcholine, two important triggers of the inflammation cascade [7]–[9]. These substances stimulate the expression of adhesion molecules and cytokines by endothelial cells, macrophages, and leukocytes; moreover, they down-regulate the endothelial nitric oxide, enhance the production of reactive oxygen species and oxidative stress, and induce endothelial cell apoptosis [10]–[12]. In addition, selective Lp-PLA2 inhibition prevented the generation of lysophosphatidylcholine and oxidized fatty acids in oxidized LDLs, which resulted in inhibition of monocyte chemotaxis and protection of macrophages against apoptotic death [7]. Hence, it is conceivable that raised Lp-PLA2 activity could predict cardiovascular (CV) events.

Consistent with this contention the West Of Scotland Coronary Prevention Study (WOSCOPS) first reported an association between increased baseline levels of Lp-PLA2 mass and risk of CV events in dyslipidemic patients [13]. This association was thereafter found in other epidemiologic studies of patients with and without prior history of CV disease [14], [15] (reviewed in [16], [17]), but was not detected in apparently healthy populations [18]–[20]. The prognostic role of either Lp-PLA2 mass or activity was found also in patients with CV disease, including high-risk patients undergoing coronary angiography [21]–[23]. Lp-PLA2 mass or activity were found to predict CV events also in patients with coronary artery disease (CAD) [24], [25], including survivors of myocardial infarction (MI) [26], and acute coronary syndromes (ACS) [27]. A meta-analysis of all the prospective studies of Lp-PLA2 performed by the Lp-PLA2 Studies Collaboration group also showed an association of both Lp-PLA2 activity and mass with a worse prognosis of CAD, ischemic stroke, and vascular mortality [28]. However, it remained uncertain if activity was better than, or equivalent to mass as a risk biomarker because a head-to-head comparison of Lp-PLA2 mass and activity was performed only in few studies. Prospective cohort studies and large meta-analysis are unlikely to be affected by selection bias and serendipitous findings. Nonetheless, an uneven distribution of variables relevant for outcome between groups can affect results even when data are analyzed with multivariate techniques owing to the limited number of covariates that can be adjusted for [29].

Thus, in the long-term prospective branch of the Genetic and Environmental factors In Coronary Artery disease (GENICA) study we tested the hypothesis that Lp-PLA2 mass and activity predict CV events by using for the first time a statistical approach that allows balancing of the groups based on known baseline determinants of CV events, thus minimizing the chances for a selection bias [30].

## Methods

### Study Participants

The protocol of the GENICA study will be briefly recalled as it was previously detailed [31], [32]. The study enrolled consecutive Caucasian patients referred for coronary angiography to investigate chest pain and/or suspected CAD between 1999 and 2001. All signed a consent form to participate in this study and the Medical Ethics Committee (Comitato Etico dell'Azienda Ospedaliera di Padova) approved the protocol. Refusal to participate was the only exclusion criterion. Information on medical history, smoking habits, presence/absence of arterial hypertension, diabetes mellitus, dyslipidemia, and current medications was gathered with a staff administered questionnaire [31], [32]. Definitions for body mass index (BMI), smoking status, diabetes mellitus, impaired glucose tolerance, hypercholesterolemia, and hypertriglyceridemia were already reported [31], [32]. Blood pressure was measured by mercury sphygmomanometer using Korotkoff phase V for diastolic, according to the WHO guidelines. Hypertension was defined as systolic blood pressure ≥140 mmHg and/or diastolic blood pressure ≥90 mmHg and/or use of antihypertensive drugs.

### Coronary angiography

Measurement of left ventricular ejection fraction (LVEF) and the grading of the CAD burden at coronary angiography (with a modified Duke Prognostic Index score)were carried out as described [33]. This score considers major epicardial coronary arteries with ≥50% diameter stenosis and goes from 0 (all major coronary arteries with lesions <50% diameter stenosis) to 100 (≥95% left main stenosis). It was shown to accurately predict 5-year mortality of medically treated patients [34].

### Laboratory measurements

All patients were studied between 8.30 a.m. and noon. Blood samples were taken immediately before coronary angiography, put on ice, and centrifuged at 3000×g (at 4°C for 10 min). Total cholesterol, HDL-cholesterol, triglycerides, glycaemia, sodium, potassium, blood urea nitrogen and creatinine levels were measured with conventional methods.

Lp-PLA2 mass and activity were assayed in a centralized way at diaDexus (San Francisco, USA) blindly with respect to the clinical data. The Lp-PLA2 concentration (mass) was measured by an ELISA method (PLAC® test, diaDexus), which is a sandwich enzyme immunoassay that uses two highly specific monoclonal antibodies for the direct measurement of Lp-PLA2 concentration. A set of calibrators is used to plot a standard curve of absorbance versus Lp-PLA2 concentration from which the Lp-PLA2 concentration in the test sample can be determined. The Lp-PLA2 activity was determined using a Colorimetric Activity Method (CAM assay, diaDexus). The assay is performed in a 96-well microplate with a colorimetric substrate that is converted, via hydrolysis, by the phospholipase enzyme. Briefly, 25 µL of standard or control samples are added per well, followed by addition of assay buffer plus substrate. The change in absorbance is immediately measured at 405 nm. The level of Lp-PLA2 activity in nmol/min/ml was calculated from the slope of the signal generated over time, based on a standard conversion factor from a *p*-Nitrophenol calibration curve.

For the Lp-PLA2 mass assay the coefficient of variation was within 3% in control samples and between 7.5% and 10% in 89% of the samples. For the Lp-PLA2 activity assay the coefficient of variation was within 2% in control samples and between 2.5% and 5% in 79–97% of the samples.

### Follow-up data

Information on the long-term outcome of the patients was gathered blindly to their biochemical profile with a predefined form through review of medical charts for the patients regularly seen at referring hospitals, and through telephone interviews of family doctors, and/or patients, and/or first-degree relatives for those not attending regular follow-up visits. Predetermined primary endpoint were CV events, including acute coronary syndromes (ACS), stroke, and CV deaths. Secondary endpoints included: ACS, acute myocardial infarction (AMI), and stroke. The endpoints were defined following the guidelines: acute myocardial infarction was defined as a typical rise and fall of biochemical markers of myocardial necrosis (troponin T or CK-MB) with at least one of the following: a) ischemic symptoms; b) development of pathologic Q waves on the ECG; c) ECG changes indicative of ischemia (ST segment elevation or depression); or d) need for coronary artery angioplasty [35]. Acute coronary syndrome was defined in the presence of an appropriate clinical setting (chest discomfort or anginal equivalent), by ECG changes indicative of ischemia and/or positive biomarkers of necrosis above the 99^th^ percentile of the upper reference limit. Congestive heart failure was defined by the presence of at least one among the following symptoms/signs, dyspnoea, ankle oedema or crepitations, and the necessity of treatment with diuretics, vasodilators, or antihypertensive drugs [36]. Stroke was defined as a neurological deficit with symptoms lasting for more than 24 h or leading to death with no apparent cause other than vascular [37]. Cardiovascular death was defined as sudden death or due to congestive heart failure, ACS, or stroke according to the Syst-Eur Trial criteria [36]. All events were validated by the adjudication committee (GPR, MZ, and GM) blinded to patients' biochemical profile.

### Statistical analysis

**Figure 1:**
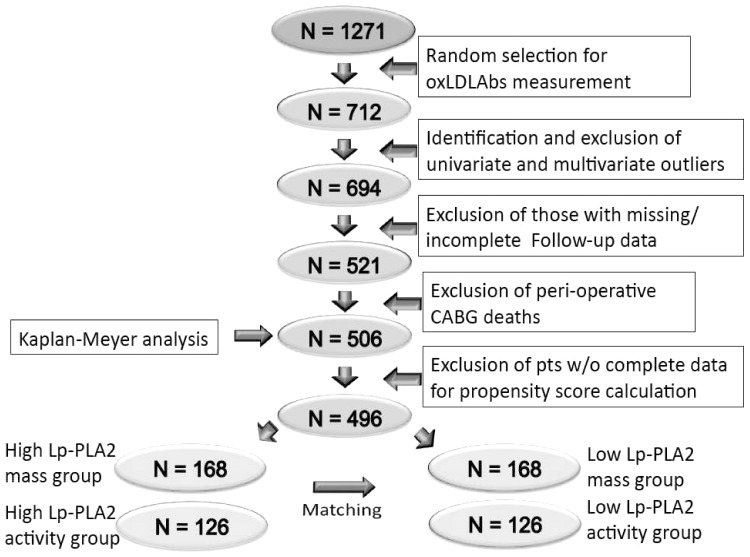
Data analysis flow chart. The flow chart shows the selection process by which the patients were submitted to statistical analysis.

Serum triglycerides, HDL- and LDL-cholesterol, age, creatinine, CAD Duke index score, LVEF, Lp-PLA2 mass and activity were examined after achievement of a Gaussian distribution by log or square root transformation, as appropriate. With single random number generation through SPSS we selected 1000 patients who had the LpPLA2 mass and activity determined, 712 of whom had significant CAD at coronary angiography (see Fig. 1). The reason for this is due to a power analysis. We had calculated that 700 patients could provide enough power to demonstrate a clinically meaningful difference in the primary endpoint. Oversizing studies usually allows demonstration of statistically significant differences that can be meaningless clinically. Furthermore, it usually results in waste of money, time and resources. Standardized z scores were calculated to identify univariate outliers and exclusion of cases with z scores exceeding |3.29|that corresponded to a p<0.001, was decided *a priori*. Mahalanobis distance was assessed by regression analysis to identify multivariate outliers; cases with c2 in excess of 32.909 (12 df at α = 0.001) were considered outliers and removed from further analysis (18 patients) [38].

Comparison of quantitative variables across groups was done by ANOVA followed by Bonferroni's *post hoc* test. Chi-square analysis was used to compare the frequencies of categorical CAD risk factors. Pearson product-moment correlation coefficient, r, was calculated to measure the association between Lp-PLA2 mass and activity.

To identify variables independently associated with Lp-PLA2 mass and activity we performed a regression analysis using inclusion and exclusion criteria of 0.05 and 0.10, respectively. The backward variable elimination was preferred to the forward inclusion because it carries a lower risk of missing relevant predictor variables. We explored the relationship between Lp-PLA2 mass and activity by using Bland and Altman plot. Given the different units of measures of the two assays we computed their standardized scores (Z-score) in order to avoid generating an artificial proportional error. Therefore, we plotted the Z-score of the Lp-PLA2 mass vs. the difference of the Lp-PLA2 activity and mass Z-scores.

The area under the receiver operating characteristic (ROC) curve was used as an estimate of diagnostic accuracy. The Youden Index (YI) ( = max (*c*) [sensitivity (*c*)+specificity (*c*)−1]) was used to identify the Lp-PLA2 mass and activity threshold values corresponding to the value of the ROC curve farthest from the identity line. This index corresponds to the optimal cutoff, defined as the value with the highest average of sensitivity and specificity. Therefore, based on the YI we divided the cohort in two groups, a high and a low Lp-PLA2 mass and activity.

Standard multiple regression analysis was used beforehand to verify the assumption that cases lost at follow up did not differ significantly from those available for survival analysis. Propensity score was calculated with logistic regression analysis including all available variables (including gender, age, BMI, LDL- and HDL-cholesterol, triglycerides, serum creatinine, homocysteine, arterial hypertension, smoking habit, LVEF, the Duke Prognostic Index of coronary atherosclerotic burden, length of follow up, history, and treatment variables) that are known to potentially affect the outcomes. To correct for the imbalance of the variables distribution between the patients with low and high Lp-PLA2 mass and activity we did a greedy matching without replacement using a caliper of 0.2 standard deviations of the logit of the propensity score [39].

The distribution of measured baseline covariates between low and high Lp-PLA2 mass and activity groups was compared between the matched samples, assessing the balance in measured variables with standardized differences (Table S1) [29]. The event occurrence with time was plotted using the Kaplan-Meier method and the survival curves for the matched set was compared with the test proposed by Klein and Moeschberger [40].

Statistical significance was defined as *P*<0.05. SPSS 18 for Windows (SPSS Italy Inc., Bologna, Italy) and MedCalc Software (Mariakerke, Belgium) were used for all analyses.

## Results

### Clinical Characteristics

According to the ATP III NCEP criteria 96% of the patients enrolled in the GENICA Study were considered to be at high CV risk, e.g. to have a 20% risk of major CV events at 10 years [41]. For this study we selected the patients with documented CAD and therefore the cohort available for this study was at even higher CV risk. The anthropometric and clinical features of the patients divided according to the Lp-PLA2 mass and activity quartiles are shown in Table 1 and 2, respectively. There were significant differences across quartiles of Lp-PLA2 mass for heart rate, total cholesterol, LDL-cholesterol, triglycerides, and LVEF. The Lp-PLA2 activity quartiles differed significantly for total cholesterol, HDL-cholesterol, LDL-cholesterol, triglycerides, homocysteine, LVEF. At variance, the CAD burden at baseline showed no association with either Lp-PLA2 mass or activity quartiles.

**Table 1:** Demographic and clinical characteristics of the subjects classified by Lp-PLA2 mass quartiles.

Quartile of Lp-PLA2 mass					
Variable	1^st^ (n = 133)	2^nd^ (n = 132)	3^rd^ (n = 132)	4^th^ (n = 132)	P =
Age (yrs)	63.14±9.01	62.64±9.34	64.47±9.76	62.39±10.40	NS
Gender (M/F %)	75.2/24.8	82.6/17.4	78.8/21.2	81.8/18.2	NS
Non-Smokers/Smokers/Ex (%)	41/16/43	35/14/51	38/8/54	32/21/47	NS
BMI (Kg/m2)	26.3±3.5	26.4±3.0	27.4±3.9	26.6±3.9	NS
Serum Creatinine (µmol/L)	90±26	89±21	95±26	96±28	NS
Serum K^+^ (mmol/L)	4.2±0.3	4.1±0.3	4.0±0.3	4.2±0.3	NS
Serum Na^+^ (mmol/L)	140±2	140±3	140±3	140±2	NS
Heart Rate (b/min)	64±9	66±10	66±10	67±9	0.037
Systolic BP, (mmHg)	134±18	135±17	135±17	132±17	NS
Diastolic BP, (mmHg)	78±9	78±10	78±8	78±9	NS
Serum Glycemia, (mmol/L)	6.3±1.9	6.4±2.1	6.2±2.0	6.1±1.6	NS
Total Cholesterol, (mg/dL)	191±37	207±43	211±38	224±47	<0.001
Mean HDL-Chol. (mg/dL)	47±12	47±11	45±10	46±11	NS
LDL Cholesterol, (mg/dL)	121±29	131±31	136±32	146±37	<0.001
Triglycerides, (mg/dL)	126.8±57.7	140.5±88.3	142.6±59.8	164.1±131.4	0.008
Homocysteine, (µmol/L)	11.3±5.2	12.5±6.9	13.2±5.8	13.3±9.1	NS
Left Ventricular EF (%)	65±12	62±12	60±14	61±15	0.049
Lp-PLA2 mass (ng/ml)	253.5±41.6	337.8±17.2	393.2±14.2	490.2±79.0	<0.001
Lp-PLA2 activity (nmol/ml/min)	91.6±20.6	105.5±19.7	119.8±20.3	136.0±24.7	<0.001
Duke CAD score	35±21	38±18	37±19	36±21	NS
Follow-up (years)	7.1±1.9	7.1±1.9	7.1±2.3	6.6±2.5	NS

Results are expressed as mean ± SD. BMI, body mass index; K^+^, potassium; Na^+^, sodium; BP, Blood Pressure; HDL, high density lipoprotein; LDL, low density lipoprotein; EF, ejection fraction; CAD, coronary artery disease.

**Table 2:** Demographic and clinical characteristics of the subjects classified by Lp-PLA2 activity quartiles.

Quartile of Lp-PLA2 activity					
Variable	1^st^ (n = 127)	2^nd^ (n = 126)	3^rd^ (n = 126)	4^th^ (n = 127)	P =
Age (yrs)	61.7±9.2	62.5±8.7	64.7±9.9	63.3±10.6	NS
Gender (M/F %)	72.4/27.6	79.4/20.6	81/19	79.8/20.2	NS
Non-Smokers/Smokers/Ex (%)	16/37/47	14/38/48	11/35/54	18/38/44	NS
BMI (Kg/m2)	26.4±3.3	26.7±3.9	26.8±3.6	27.3±3.9	NS
Serum Creatinine (µmol/L)	89±26	92±22	92±26	95±27	NS
Serum K^+^ (mmol/L)	4.1±0.3	4.2±0.3	4.2±0.3	4.2±0.3	NS
Serum Na^+^ (mmol/L)	140±3	139±3	140±3	140±3	NS
Heart Rate (b/min)	64±9	66±10	66±9	67±9	NS
Systolic BP, (mmHg)	134±17	135±17	134±19	133±17	NS
Diastolic BP, (mmHg)	78±9	78±9	78±9	78±9	NS
Serum Glycemia, (mmol/L)	6.4±1.8	6.3±2.1	6.1±1.8	6.2±1.9	NS
Total Cholesterol, (mg/dL)	194±42	208±37	213±41	218±44	<0.001
Mean HDL-Chol. (mg/dL)	48±13	47±10	46±11	43±10	<0.001
LDL Cholesterol, (mg/dL)	121±31	134±31	136±34	142±35	<0.001
Triglycerides, (mg/dL)	125±70	136±61	145±79	158±79	<0.001
Homocysteine, (µmol/L)	11.5±7.0	11.4±4.3	12.4±5.5	14.6±9.3	<0.001
Left Ventricular EF (%)	65±12	64±12	60±13	61±15	0.037
Lp-PLA2 mass (ng/ml)	294.3±71.5	359.9±75.4	392.3±82.0	442.3±96.9	<0.001
Lp-PLA2 activity (nmol/ml/min)	80.7±11.6	104.1±4.9	121.0±5.5	148.7±16	<0.001
Duke CAD score	34±20	37±19	37±20	37±20	NS
Follow-up (years)	7.1±1.7	7.0±2.3	6.9±2.3	6.7±2.4	NS

Results are expressed as mean ± SD. BMI, body mass index; K^+^, potassium; Na^+^, sodium; BP, Blood Pressure; HDL, high density lipoprotein; LDL, low density lipoprotein; EF, ejection fraction; CAD, coronary artery disease.

Linear regression analysis showed that predictors of the Lp-PLA2 mass were HDL (ß = −0.163), serum creatinine (ß = 0.191), and LDL (ß = 0.302) plasma levels (adjusted R^2^ = 0.125, F = 13.96, p<0.0001) (Table 3), and that significant predictors of Lp-PLA2 activity were gender (ß = −0.122), HDL cholesterol (ß = −0.228), LDL cholesterol (ß = 0.280), and homocysteinemia (ß = 0.116) (adjusted R^2^ = 0.13, F = 11.12, p<0.0001) (Table 3). The overall use of lipid-lowering drugs was small in the GENICA study as reported [42]. Of the 529 patients with available Lp-PLA2 mass determination only 182 (34%) were on statins at enrolment; they had lower Lp-PLA2 mass than those not on statins (338.6±7.3 vs. 376.4±5.5 ng/ml, p<0.0001). Of the 506 patients with available Lp-PLA2 activity determination only 173 (34%) were on statins at enrollment; they also had lower Lp-PLA2 activity than those not on statins (108.2±27.2 vs. 116.4±26.51 ng/ml, p<0.0001).

**Table 3:** Stepwise linear regression analysis of determinants of Lp-PLA2 mass and activity.

Lp-PLA2 Mass		
Variables in the model	β	P =
HDL-cholesterol	−0.163	0.007
LDL-cholesterol	0.302	0.001
Creatinine	0.191	0.001
***Overall model statistics*** *Adjusted R^2^ = 0.125 F = 13.96 p<0.0001*		

Lp-PLA2 Activity		
Variables in the model	β	P
Gender	−0.122	0.039
HDL-cholesterol	−0.228	0.001
LDL-cholesterol	0.280	0.001
Homocysteinemia	0.116	0.043
***Overall model statistics*** *Adjusted R^2^ = 0.130 F = 11.12 p<0.0001*		

Significant predictors of Lp-PLA2 mass were creatinine, HDL- and LDL. Significant predictors of Lp-PLA2 activity were gender, HDL- and LDL-cholesterol, and homocysteinemia.

### Relationship between Lp-PLA2 mass and activity

There was a moderate positive linear relationship between Lp-PLA2 mass and activity (r = 0.60, p<0.0001). Increasing levels of the Lp-PLA2 mass (Z-scores) corresponded to more negative values of difference between Lp-PLA2 activity and mass and vice versa (Fig. S1). Moreover, the most extremes Lp-PLA2 mass Z-score values fell outside the 95% confidence interval. These results imply that the proportion of active Lp-PLA2 enzyme varies much less than that of Lp-PLA2 mass and, conversely, that the biological effects of the enzyme are only partially accounted for by its circulating mass. These findings might therefore explain the prognostic value on CV events of plasma Lp-PLA2 activity as well as the lack of prognostic value of the Lp-PLA2 mass.

### Follow up data

**Figure 2:**
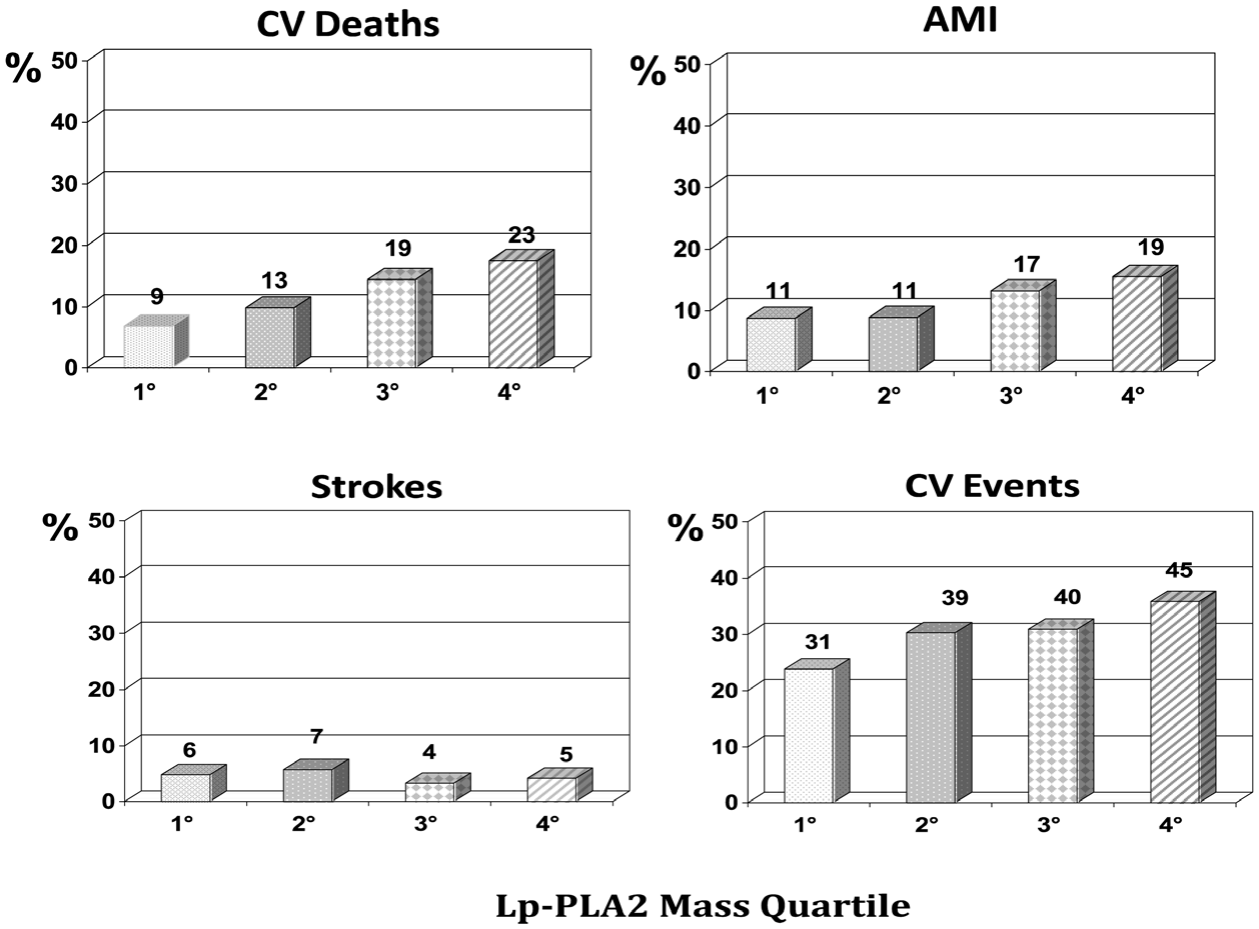
Cardiovascular events by Lp-PLA2 mass. The bar graphs show cardiovascular death and events rate by quartiles of Lp-PLA2 mass (the absolute number of events is shown above each column). Cardiovascular deaths (p = 0.020) were significantly different across Lp-PLA2 mass quartiles. AMI: acute myocardial infarction.

**Figure 3:**
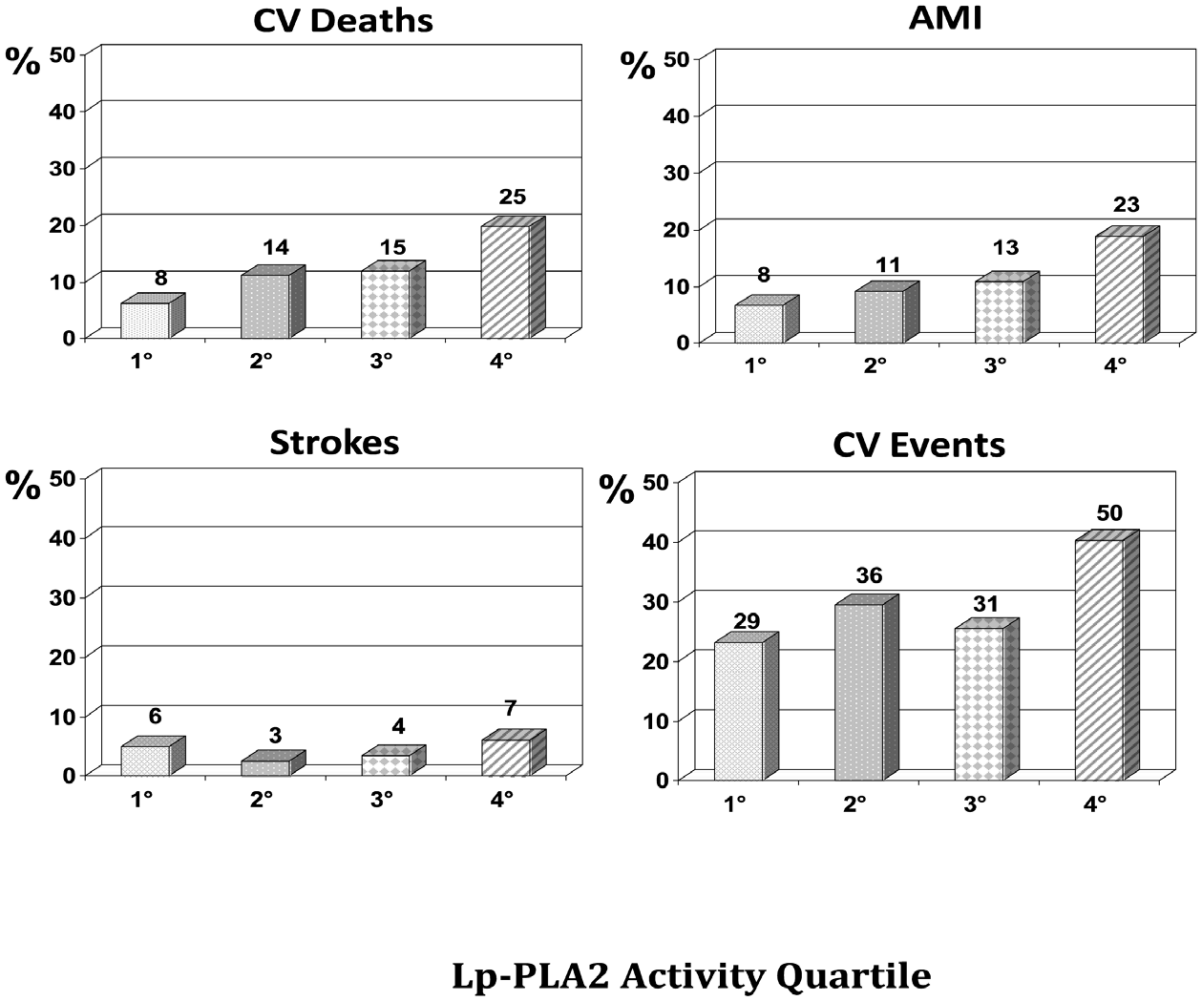
Cardiovascular events by Lp-PLA2 activity. The bar graphs show cardiovascular death and events rate by quartiles of Lp-PLA2 activity (the absolute number of events is shown above each column). Cardiovascular deaths (p = 0.012), events (p = 0.016), acute myocardial infarction (AMI) (p = 0.019) were significantly different across Lp-PLA2 activity quartiles.

After exclusion of patients with incomplete follow-up data and of multivariate outliers according to the procedure of Tabachnick and Fidell [38], full data were available for 521 (75%) patients. Fifteen cases of perioperative death related to coronary artery by-pass surgery or its complications were also excluded from the analysis. Thus, the final analysis was carried out in 506 patients. The median length of follow-up was 7.2 years (range 1–12.7 years) during which 62 (12.3%) CV deaths and 129 (25.5%) CV events were observed. The CV death and event rate according to the Lp-PLA2 mass and activity quartiles (Fig. 2 and 3, respectively) evidenced a stepwise increase of CV deaths (p = 0.020 and p = 0.012, respectively), CV events (p = 0.016 for Lp-PLA2 activity), and AMI (p = 0. 019 for Lp-PLA2 activity), but not for strokes, for either variable. However, it has to be noted that only 22 and 20 strokes were observed at follow up, for Lp-PLA2 mass and activity, respectively, in this cohort of CAD patients.

### Receiver operating characteristic curve analysis

The YI cut-off value that best predicted CV events for Lp-PLA2 mass was 405.9 ng/ml(AUC 0.551, 95% confidence interval (CI) 0.507–0.595, p = 0.076). For Lp-PLA2 activity the corresponding YI was 136.1 nmol/ml/min (AUC 0.707, 95% CI 0.663–0.749, p<0.0001). Therefore we used the YI values for our predefined primary endpoint to split our cohort into a high and a low Lp-PLA2 mass and activity, which were thereafter analyzed in terms of death and CV events free survival. The concordance between Lp-PLA2 mass and activity for classification into a high vs. a low level group was moderate (kappa = 0.40, p<0.0001).

### Lipoprotein associated-PLA2 mass and activity survival analysis at Kaplan-Meier

At Kaplan-Meier analysis the group with high Lp-PLA2 mass had worse CV-death-free survival (80.6% vs. 91.2, respectively, p<0.0001), CV event-free survival (65.6% vs. 77.0%, respectively, p = 0.008), ACS-free survival (75.2% vs. 84.6%, respectively, p = 0.011), and AMI-free survival (82.7% vs. 91.1%, respectively, p = 0.005) compared to the low Lp-PLA2 mass. The patients in the high Lp-PLA2 activity group also had worse CV-death-free survival (77.6% vs. 90.2%, respectively, p = 0.001), CV event-free survival (63.5% vs. 76.4%, respectively, p = 0.011), ACS-free survival (76.0% vs. 84.3%, respectively, p = 0.043), and AMI-free survival (81.6% vs. 91.1%, respectively, p = 0.004)than those in the low Lp-PLA2 activity group.

### Survival analysis after propensity score matching

We used a propensity score matching analysis to rule out the possibility that the association of Lp-PLA2 mass and activity with worse survival rates were due to an unbalanced distribution of confounding variables between the high and the low Lp-PLA2 mass and activity groups.

This analysis was feasible for 496 patients who had complete data for all the variables necessary to compute the propensity score. The high Lp-PLA2 mass group comprised 203 patients and, among these, 83% (n = 168) of patients could be matched by propensity score with an equal number in the low Lp-PLA2 mass group. Overall 24 (7.1%) CV deaths and 73 (21.7%) CV events were observed in these 336 patients. The patients in the high Lp-PLA2 mass group tended to have a worse CV death- (91.1% vs. 94.6%, p = NS) and event-free survival (75.0% vs. 80.5%, p = NS) than the low Lp-PLA2 mass group, but the differences were not statistically significant.

**Figure 4:**
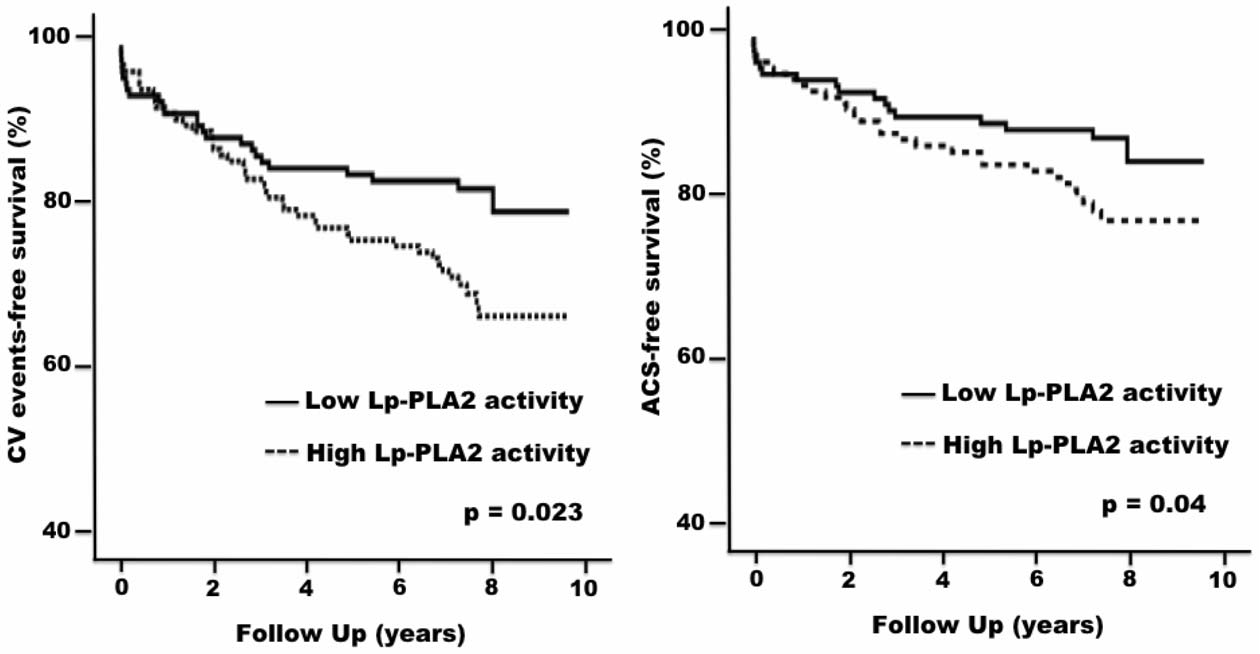
Cardiovascular events- and acute coronary syndromes-free survival. Kaplan-Meier curves show cardiovascular (CV) events- (left Panel) and acute coronary syndromes (ACS)-free survival (right Panel) in the propensity score-matched high-risk patients divided into the high and low Lp-PLA2 activity group. Patients with high Lp-PLA2 activity had a significantly lower CV events-free survival and a significantly lower ACS-free survival.

Of the patients in the high Lp-PLA2 activity group 94% (n = 126) could be matched with an equal number in the low Lp-PLA2 activity group. Compared to the patients in the low Lp-PLA2 activity group those in the high Lp-PLA2 activity group showed a trend toward worse CV death- (81.4% vs. 91.6%, p = 0.1) and AMI-free survival (81.2% vs. 91.5%, p = 0.07), but significantly worse CV event- (66.7% vs. 79.5%, p = 0.023) and ACS event-free survival (75.4% vs. 85.6%, p = 0.04) (Figure 4). These results were substantially similar to those obtained at Cox regression analysis (data S1).

## Discussion

After the report that Lp-PLA2 mass in the WOSCOPS Study was directly associated with a worse CV prognosis in dyslipidemic patients [13], a prognostic role of Lp-PLA2 has been suggested by several studies that, however, paid little attention to many potential confounding factors. For example, the direct association of Lp-PLA2 mass and activity with the atherogenic LDL fraction, and the inverse correlation with the “protective” HDL-cholesterol fraction could explain, at least in part, the adverse prognostic role of Lp-PLA2 that was observed.

This study is novel in that it investigated patients unequivocally at very high CV risk in which demonstration of the prognostic role of novel markers could be most difficult. Moreover, this study exploited for the first time in the field of Lp-PLA2 research use of the propensity score matching analysis, a technique that allows comparison of survival between groups after individual matching of the patients in each group for all potentially relevant variables. At variance with classical multivariate Cox's regression analysis where the number of covariates that can be included in the model is limited, this score took into due account most potentially relevant variables, including lipoprotein fractions, ongoing treatment with lipid-lowering agents and also the coronary atherosclerotic burden. It is, therefore, interesting to find that a high Lp-PLA2 activity was associated with a worse CV prognosis, both in terms of major CV events and of acute coronary syndromes (Fig. 4), independent from all major CV risk factors, as shown by the consistent results of univariate Kaplan-Meier analysis and of the propensity score matching. Thus, with fresh novel long-term data the present findings lend support to those of studies in different patients populations (reviewed in [16], [17]) and to the conclusions of a large meta-analysis [28], which incorporated a much shorter follow-up, and therefore a much smaller number of CV events in this cohort.

The propensity score matching analysis confirmed the prognostic role of Lp-PLA2 found at univariate Kaplan-Meier analysis only for activity and not for mass, even though a larger number of patients could be matched for Lp-PLA2 mass than for Lp-PLA2 activity. Our results were opposite to those reported by Cook et al. that Lp-PLA2 mass and not activity was associated to CV events in postmenopausal women [15]. This difference is probably due to the tight correlation of Lp-PLA2 activity with HDL, as reported by the authors, which might have introduced a collinearity problem in their Cox regression analysis. Brilakis et al. also found an independent association between Lp-PLA2 mass and CV events in patients undergoing coronary angiography after a shorter median follow up [21], but they did not support their finding by a propensity score matching analysis. Hence, while it is likely that propensity score matching analysis can provide more robust results than univariate analysis, our data show that the activity of the Lp-PLA2 enzyme in the plasma, rather than its simple detection as mass, conveys prognostic information. The strength of this conclusion is supported by our use of a head-to-head comparison of the two assays that were performed in a way totally blinded to the clinical data and centralized in the same laboratory at diaDexus. There are several potential explanations why Lp-PLA2 activity can predicts outcome more accurately that Lp-PLA2 mass. The former reflects the biological function of the enzyme, and probably macrophage activation in unstable atherosclerotic plaques, better than the latter. Moreover, at Bland and Altman plot (Fig. S1) it appeared that there was a proportional error that affected the agreement between the assays for the most extreme Lp-PLA2 mass values. Regardless of the explanation, these results have sound practical implications for the selection of the type of Lp-PLA2 assay to be chosen for the risk stratification purposes in clinical practice.

Another interesting finding, which extends previous findings on Lp-PLA2 mass [21], is the lack of association of Lp-PLA2 mass and activity with the angiographically determined CAD burden. This result, which might seem to contrast with the prognostic role of Lp-PLA2 activity, could be explained on the ground of the different determinants of plaque destabilization and plaque growth [43]. It is worth remembering in this context that the culprit vulnerable plaque causing acute coronary syndromes commonly occurs at sites determining stenosis <50% [44], [45]. The view that the CAD atherosclerotic burden can be a poor surrogate for coronary plaque destabilization and coronary events is also supported by recent findings on Lp-PLA2 activity. Using virtual histology intravascular ultrasound no differences in atheroma volume were found between 330 patients with angiographically documented CAD randomized to the selective Lp-PLA2 inhibitor darapladib or to placebo [46]. However, darapladib inhibited the increase of necrotic core volume, an index of plaque instability. Thus, available data from prospective cohort observational studies and from the only randomized clinical trial published thus far are consistent with an involvement of Lp-PLA2 activity in the progression of coronary atherosclerosis leading to coronary events.

Of further interest our study evidenced in these high-risk patients an association of homocysteine with Lp-PLA2 activity, but not Lp-PLA2 mass, a finding that is in line with those of a much smaller study performed by Atik et al. [47] in 42 patients undergoing elective carotid endoatherectomy. It might be contended that this direct relationship is explained by considering that a) Lp-PLA2 activity is markedly reduced *in vivo* when the enzyme is bound to HDL [48], b) a methionine-rich diet, which raises homocysteine, has a blunting effect on HDL production in mice [49]. However, since the Lp-PLA2 activity assay is carried out after the enzyme is completely released from its lipoprotein carrier, and therefore, measured Lp-PLA2 activity is similar regardless of which particle the Lp-PLA2 is trafficking with, this explanation is in our view unlikely.

### Limitations and strengths of the study

Some potential limitations must be underscored: it could be argued that the 25% rate of patients lost at follow up have biased our results. However, considering the length of our follow up this drop-out rate is comparable to that found in similar studies [50], [51]. Moreover, it seems unlikely that a selection bias affected our results and conclusions owing to the fact that the cases lost did not differ significantly for any variables at baseline from those available at follow up.

The GENICA study enrolled patients with coronary artery disease who, therefore, had a rather small number of strokes at follow-up. Hence, it is likely that the study was underpowered to show a prognostic effect of Lp-PLA2 activity and mass on stroke. Finally, albeit the study size might seem small at first look, matching as many as 252 and 336 patients for Lp-PLA2 activity and mass, respectively, with the propensity score for all the relevant variables should be seen as a relevant accomplishment. Hence, these limitations are outweighed by several strengths including the head-to-head comparison of Lp-PLA2 mass and activity assayed in a centralized way, the long-term follow-up, the comprehensive information on anthropometric, biochemical, and clinical features at baseline, and an accurate assessment of CV events at follow-up.

### Conclusions

Lipoprotein-PLA2 activity, but not Lp-PLA2 mass, predicts cardiovascular events and acute coronary syndromes in high-risk Caucasian patients with coronary artery disease. Moreover, the prognostic information conveyed by plasma Lp-PLA2 activity was independent of and additive to that of other common major cardiovascular risk factors and also of coronary atherosclerotic burden. Hence, these data implicate Lp-PLA2 activity in atherosclerotic plaque erosion and rupture, a contention supported also by the results of a pilot randomized controlled trial [46]. Two ongoing clinical trials involving the use of thedarapladibLp-PLA2 inhibitor, the STABILITY [52] and the SOLID-TIMI 52 [53] trials are expected to provide further proof of Lp-PLA2 role in progression and/or destabilization of the atherosclerotic plaque.

## Supporting Information

Figure S1 **Lp-PLA2 Z-scores Bland and Altman plot.** Increasing Lp-PLA2 mass z-score is associated with more negative values of the difference between Lp-PLA2 activity and mass and vice versa. The most extremes Lp-PLA2 mass Z-score values fell outside the confidence interval. **Methods**. We explored the relationship between lipoprotein-associated phospholipase A2 (Lp-PLA2) mass and activity by using Bland and Altman plot. Given the different units of measures of the two assays we computed their standardized scores (Z-score) in order to avoid generating an artificial proportional error. The z-score of the Lp-PLA2 mass vs. the difference of the Lp-PLA2 activity and mass z-scores was therefore plotted. Results. As shown in the supplemental figure, increasing levels of the Lp-PLA2 mass z-score corresponded to more negative values of the difference between Lp-PLA2 activity and mass and vice versa. Moreover, the most extremes Lp-PLA2 mass Z-score values fell outside the confidence interval. These results indicate that the proportion of the active counterpart of Lp-PLA2 varies much less than that of Lp-PLA2 mass and therefore that he biological effects of the enzyme are only partially accounted for by its circulating mass. These findings might explain why plasma Lp-PLA2 activity and not mass had prognostic value on cardiovascular events.
(PPTX)

Table S1 **Standardized differences of low and high Lp-PLA2 activity after matching.**
(DOC)

Data S1 Lp-PLA2 mass and activity Cox's regression analyses.
(DOC)

## References

[1] Asano K, Okamoto S, Fukunaga K, Shiomi T, Mori T, et al (1999) Cellular source(s) of platelet-activating-factor acetylhydrolase activity in plasma. Biochemical and Biophysical Research Communications 261: 511–514. 10425216 10.1006/bbrc.1999.1066

[2] Burke JE, Dennis EA (2009) Phospholipase A2 biochemistry. Cardiovascular Drugs and Therapy/Sponsored by the International Society of Cardiovascular Pharmacotherapy 23: 49–59. 10.1007/s10557-008-6132-9 PMC2823292 18931897

[3] Gaubatz JW, Gillard BK, Massey JB, Hoogeveen RC, Huang M, et al (2007) Dynamics of dense electronegative low density lipoproteins and their preferential association with lipoprotein phospholipase A(2). Journal of Lipid Research 48: 348–357. 17102149 10.1194/jlr.M600249-JLR200

[4] Stafforini DM, McIntyre TM, Carter ME, Prescott SM (1987) Human plasma platelet-activating factor acetylhydrolase. association with lipoprotein particles and role in the degradation of platelet-activating factor. The Journal of Biological Chemistry 262: 4215–4222. 3549727

[5] Hakkinen T, Luoma JS, Hiltunen MO, Macphee CH, Milliner KJ, et al (1999) Lipoprotein-associated phospholipase A(2), platelet-activating factor acetylhydrolase, is expressed by macrophages in human and rabbit atherosclerotic lesions. Arteriosclerosis, Thrombosis, and Vascular Biology 19: 2909–2917. 10.1161/01.atv.19.12.2909 10591668

[6] Kolodgie FD, Burke AP, Skorija KS, Ladich E, Kutys R, et al (2006) Lipoprotein-associated phospholipase A2 protein expression in the natural progression of human coronary atherosclerosis. Arteriosclerosis, Thrombosis, and Vascular Biology 26: 2523–2529. 10.1161/01.ATV.0000244681.72738.bc 16960105

[7] MacPhee CH, Moores KE, Boyd HF, Dhanak D, Ife RJ, et al (1999) Lipoprotein-associated phospholipase A2, platelet-activating factor acetylhydrolase, generates two bioactive products during the oxidation of low-density lipoprotein: Use of a novel inhibitor. The Biochemical Journal 338 Pt 2: 479–487. 10024526 PMC1220076

[8] Kume N, Cybulsky MI, Gimbrone MA Jr (1992) Lysophosphatidylcholine, a component of atherogenic lipoproteins, induces mononuclear leukocyte adhesion molecules in cultured human and rabbit arterial endothelial cells. The Journal of Clinical Investigation 90: 1138–1144. 1381720 10.1172/JCI115932 PMC329976

[9] Kohno M, Yokokawa K, Yasunari K, Minami M, Kano H, et al (1998) Induction by lysophosphatidylcholine, a major phospholipid component of atherogenic lipoproteins, of human coronary artery smooth muscle cell migration. Circulation 98: 353–359. 9711941 10.1161/01.cir.98.4.353

[10] Inoue N, Takeshita S, Gao D, Ishida T, Kawashima S, et al (2001) Lysophosphatidylcholine increases the secretion of matrix metalloproteinase 2 through the activation of NADH/NADPH oxidase in cultured aortic endothelial cells. Atherosclerosis 155: 45–52. 11223425 10.1016/s0021-9150(00)00530-x

[11] Safaya R, Chai H, Kougias P, Lin P, Lumsden A, et al (2005) Effect of lysophosphatidylcholine on vasomotor functions of porcine coronary arteries. The Journal of Surgical Research 126: 182–188. 15919417 10.1016/j.jss.2005.01.015

[12] Takahashi M, Okazaki H, Ogata Y, Takeuchi K, Ikeda U, et al (2002) Lysophosphatidylcholine induces apoptosis in human endothelial cells through a p38-mitogen-activated protein kinase-dependent mechanism. Atherosclerosis 161: 387–394. 11888522 10.1016/s0021-9150(01)00674-8

[13] Packard CJ, O'Reilly DS, Caslake MJ, McMahon AD, Ford I, et al (2000) Lipoprotein-associated phospholipase A2 as an independent predictor of coronary heart disease. west of scotland coronary prevention study group. The New England Journal of Medicine 343: 1148–1155. 11036120 10.1056/NEJM200010193431603

[14] Kizer JR, Umans JG, Zhu J, Devereux RB, Wolfert RL, et al (2012) Lipoprotein-associated phospholipase A(2) mass and activity and risk of cardiovascular disease in a population with high prevalences of obesity and diabetes: The strong heart study. Diabetes Care 35: 840–847. 22338104 10.2337/dc11-1639 PMC3308309

[15] Cook NR, Paynter NP, Manson JE, Martin LW, Robinson JG, et al (2012) Clinical utility of lipoprotein-associated phospholipase A2 for cardiovascular disease prediction in a multiethnic cohort of women. Clinical Chemistry 58: 1352–1363. 22859728 10.1373/clinchem.2012.188870 PMC3621122

[16] Anderson JL (2008) Lipoprotein-associated phospholipase A2: An independent predictor of coronary artery disease events in primary and secondary prevention. The American Journal of Cardiology 101: 23F–33F. 10.1016/j.amjcard.2008.04.015 18549868

[17] Epps KC, Wilensky RL (2011) Lp-PLA- a novel risk factor for high-risk coronary and carotid artery disease. Journal of Internal Medicine 269: 94–106. 21054587 10.1111/j.1365-2796.2010.02297.x

[18] Blake GJ, Dada N, Fox JC, Manson JE, Ridker PM (2001) A prospective evaluation of lipoprotein-associated phospholipase A(2) levels and the risk of future cardiovascular events in women. Journal of the American College of Cardiology 38: 1302–1306. 11691499 10.1016/s0735-1097(01)01554-6

[19] Ballantyne CM, Hoogeveen RC, Bang H, Coresh J, Folsom AR, et al (2004) Lipoprotein-associated phospholipase A2, high-sensitivity C-reactive protein, and risk for incident coronary heart disease in middle-aged men and women in the atherosclerosis risk in communities (ARIC) study. Circulation 109: 837–842. 14757686 10.1161/01.CIR.0000116763.91992.F1

[20] Rana JS, Arsenault BJ, Despres JP, Cote M, Talmud PJ, et al (2011) Inflammatory biomarkers, physical activity, waist circumference, and risk of future coronary heart disease in healthy men and women. European Heart Journal 32: 336–344. 19224930 10.1093/eurheartj/ehp010

[21] Brilakis ES, McConnell JP, Lennon RJ, Elesber AA, Meyer JG, et al (2005) Association of lipoprotein-associated phospholipase A2 levels with coronary artery disease risk factors, angiographic coronary artery disease, and major adverse events at follow-up. European Heart Journal 26: 137–144. 15618069 10.1093/eurheartj/ehi010

[22] May HT, Horne BD, Anderson JL, Wolfert RL, Muhlestein JB, et al (2006) Lipoprotein-associated phospholipase A2 independently predicts the angiographic diagnosis of coronary artery disease and coronary death. American Heart Journal 152: 997–1003. 17070179 10.1016/j.ahj.2006.01.011

[23] Winkler K, Hoffmann MM, Winkelmann BR, Friedrich I, Schafer G, et al (2007) Lipoprotein-associated phospholipase A2 predicts 5-year cardiac mortality independently of established risk factors and adds prognostic information in patients with low and medium high-sensitivity C-reactive protein (the ludwigshafen risk and cardiovascular health study). Clinical Chemistry 53: 1440–1447. 17573419 10.1373/clinchem.2007.086298

[24] Koenig W, Twardella D, Brenner H, Rothenbacher D (2006) Lipoprotein-associated phospholipase A2 predicts future cardiovascular events in patients with coronary heart disease independently of traditional risk factors, markers of inflammation, renal function, and hemodynamic stress. Arteriosclerosis, Thrombosis, and Vascular Biology 26: 1586–1593. 10.1161/01.ATV.0000222983.73369.c8 16627803

[25] Sabatine MS, Morrow DA, O'Donoghue M, Jablonksi KA, Rice MM, et al (2007) Prognostic utility of lipoprotein-associated phospholipase A2 for cardiovascular outcomes in patients with stable coronary artery disease. Arteriosclerosis, Thrombosis, and Vascular Biology 27: 2463–2469. 10.1161/ATVBAHA.107.151670 17766330

[26] Gerber Y, McConnell JP, Jaffe AS, Weston SA, Killian JM, et al (2006) Lipoprotein-associated phospholipase A2 and prognosis after myocardial infarction in the community. Arteriosclerosis, Thrombosis, and Vascular Biology 26: 2517–2522. 10.1161/01.ATV.0000240406.89440.0c 16902161

[27] O'Donoghue M, Morrow DA, Sabatine MS, Murphy SA, McCabe CH, et al (2006) Lipoprotein-associated phospholipase A2 and its association with cardiovascular outcomes in patients with acute coronary syndromes in the PROVE IT-TIMI 22 (PRavastatin or atorVastatin evaluation and infection therapy-thrombolysis in myocardial infarction) trial. Circulation 113: 1745–1752. 16537575 10.1161/CIRCULATIONAHA.105.612630

[28] Lp-PLA(2) Studies Collaboration (2010) Thompson A, Gao P, Orfei L, Watson S, et al (2010) Lipoprotein-associated phospholipase A(2) and risk of coronary disease, stroke, and mortality: Collaborative analysis of 32 prospective studies. Lancet 375: 1536–1544. 20435228 10.1016/S0140-6736(10)60319-4 PMC2864403

[29] Heinze G, Juni P (2011) An overview of the objectives of and the approaches to propensity score analyses. European Heart Journal 32: 1704–1708. 21362706 10.1093/eurheartj/ehr031

[30] Rosenbaum P.R RDB (1984) Reducing bias in observational studies using subclassification on the propensity score. J Am Stat Assoc 79: 516–524.

[31] Rossi GP, Cesari M, De Toni R, Zanchetta M, Maiolino G, et al (2003) Antibodies to oxidized low-density lipoproteins and angiographically assessed coronary artery disease in white patients. Circulation 108: 2467–2472. 14581399 10.1161/01.CIR.0000097122.19430.48

[32] Rossi GP, Cesari M, Zanchetta M, Colonna S, Maiolino G, et al (2003) The T-786C endothelial nitric oxide synthase genotype is a novel risk factor for coronary artery disease in caucasian patients of the GENICA study. Journal of the American College of Cardiology 41: 930–937. 12651036 10.1016/s0735-1097(02)03012-7

[33] Cesari M, Pessina AC, Zanchetta M, De Toni R, Avogaro A, et al (2006) Low plasma adiponectin is associated with coronary artery disease but not with hypertension in high-risk nondiabetic patients. Journal of Internal Medicine 260: 474–483. 17040254 10.1111/j.1365-2796.2006.01714.x

[34] Califf RM, Armstrong PW, Carver JR, D'Agostino RB, Strauss WE (1996) 27th bethesda conference: Matching the intensity of risk factor management with the hazard for coronary disease events. task force 5. stratification of patients into high, medium and low risk subgroups for purposes of risk factor management. Journal of the American College of Cardiology 27: 1007–1019. 8609316 10.1016/0735-1097(96)87733-3

[35] Alpert JS, Thygesen K, Antman E, Bassand JP (2000) Myocardial infarction redefined–a consensus document of the joint european society of Cardiology/American college of cardiology committee for the redefinition of myocardial infarction. Journal of the American College of Cardiology 36: 959–969. 10987628 10.1016/s0735-1097(00)00804-4

[36] Staessen JA, Fagard R, Thijs L, Celis H, Arabidze GG, et al (1997) Randomised double-blind comparison of placebo and active treatment for older patients with isolated systolic hypertension. the systolic hypertension in europe (syst-eur) trial investigators. Lancet 350: 757–764. 9297994 10.1016/s0140-6736(97)05381-6

[37] Thorvaldsen P, Asplund K, Kuulasmaa K, Rajakangas AM, Schroll M (1995) Stroke incidence, case fatality, and mortality in the WHO MONICA project. world health organization monitoring trends and determinants in cardiovascular disease. Stroke; a Journal of Cerebral Circulation 26: 361–367. 10.1161/01.str.26.3.361 7886707

[38] Tabachnick BG, Fidell LS, editors. (2001) Using multivariate statistics. : Allyn & Bacon, Boston, MA. 56–110 p.

[39] Austin PC (2009) Some methods of propensity-score matching had superior performance to others: Results of an empirical investigation and monte carlo simulations. Biometrical Journal.Biometrische Zeitschrift 51: 171–184. 19197955 10.1002/bimj.200810488

[40] Klein J.P MML. (1997) Survival analysis: Techniques for censored and truncated data. : New York: Springer Verlag.

[41] Cesari M, Maiolino G, Colonna S, Zanchetta M, Pedon L, et al (2003) Under treatment with lipid-lowering drugs of high-risk coronary heart disease patients of the GENICA study. Journal of Cardiovascular Pharmacology 42: 484–490. 14508233 10.1097/00005344-200310000-00005

[42] Cesari M, Maiolino G, Colonna S, Zanchetta M, Pedon L, et al (2003) Under treatment with lipid-lowering drugs of high-risk coronary heart disease patients of the GENICA study. Journal of Cardiovascular Pharmacology 42: 484–490. 14508233 10.1097/00005344-200310000-00005

[43] Rossi GP, Maiolino G, Zanchetta M, Sticchi D, Pedon L, et al (2006) The T(-786)C endothelial nitric oxide synthase genotype predicts cardiovascular mortality in high-risk patients. Journal of the American College of Cardiology 48: 1166–1174. 16979000 10.1016/j.jacc.2006.05.046

[44] Ambrose JA, Tannenbaum MA, Alexopoulos D, Hjemdahl-Monsen CE, Leavy J, et al (1988) Angiographic progression of coronary artery disease and the development of myocardial infarction. Journal of the American College of Cardiology 12: 56–62. 3379219 10.1016/0735-1097(88)90356-7

[45] Little WC, Constantinescu M, Applegate RJ, Kutcher MA, Burrows MT, et al (1988) Can coronary angiography predict the site of a subsequent myocardial infarction in patients with mild-to-moderate coronary artery disease? Circulation 78: 1157–1166. 3180375 10.1161/01.cir.78.5.1157

[46] Serruys PW, Garcia-Garcia HM, Buszman P, Erne P, Verheye S, et al (2008) Effects of the direct lipoprotein-associated phospholipase A(2) inhibitor darapladib on human coronary atherosclerotic plaque. Circulation 118: 1172–1182. 18765397 10.1161/CIRCULATIONAHA.108.771899

[47] Atik B, Johnston SC, Dean D (2010) Association of carotid plaque lp-PLA(2) with macrophages and chlamydia pneumoniae infection among patients at risk for stroke. PloS One 5: e11026. 20543948 10.1371/journal.pone.0011026 PMC2882946

[48] Gazi I, Lourida ES, Filippatos T, Tsimihodimos V, Elisaf M, et al (2005) Lipoprotein-associated phospholipase A2 activity is a marker of small, dense LDL particles in human plasma. Clinical Chemistry 51: 2264–2273. 16223884 10.1373/clinchem.2005.058404

[49] Velez-Carrasco W, Merkel M, Twiss CO, Smith JD (2008) Dietary methionine effects on plasma homocysteine and HDL metabolism in mice. The Journal of Nutritional Biochemistry 19: 362–370. 17707632 10.1016/j.jnutbio.2007.05.005 PMC2430472

[50] Koh JS, Park JH, Shin DH, Youn TJ, Oh IY, et al (2012) Risk factors and effects on long-term outcomes of cardiac troponin I elevation after drug-eluting stent implantation in patients with stable coronary artery disease. The American Journal of Cardiology 109: 461–465. 22133750 10.1016/j.amjcard.2011.09.039

[51] Hochman JS, Reynolds HR, Dzavik V, Buller CE, Ruzyllo W, et al (2011) Long-term effects of percutaneous coronary intervention of the totally occluded infarct-related artery in the subacute phase after myocardial infarction. Circulation 124: 2320–2328. 22025606 10.1161/CIRCULATIONAHA.111.041749 PMC3235739

[52] White H, Held C, Stewart R, Watson D, Harrington R, et al (2010) Study design and rationale for the clinical outcomes of the STABILITY trial (STabilization of atherosclerotic plaque by initiation of darapLadIb TherapY) comparing darapladib versus placebo in patients with coronary heart disease. American Heart Journal 160: 655–661. 20934559 10.1016/j.ahj.2010.07.006

[53] O'Donoghue ML, Braunwald E, White HD, Serruys P, Steg PG, et al (2011) Study design and rationale for the stabilization of pLaques usIng darapladib-thrombolysis in myocardial infarction (SOLID-TIMI 52) trial in patients after an acute coronary syndrome. American Heart Journal 162: 613–619.e1. 21982651 10.1016/j.ahj.2011.07.018

