# Supplementary figures and images for: Lipoprotein-Associated Phospholipase A2 Activity Predicts Cardiovascular Events in High Risk Coronary Artery Disease Patients
Source: PLoS One. 2012 Oct 31;7(10):e48171. doi: 10.1371/journal.pone.0048171 (PMC3485195; doi:10.1371/journal.pone.0048171)

## Slide 1
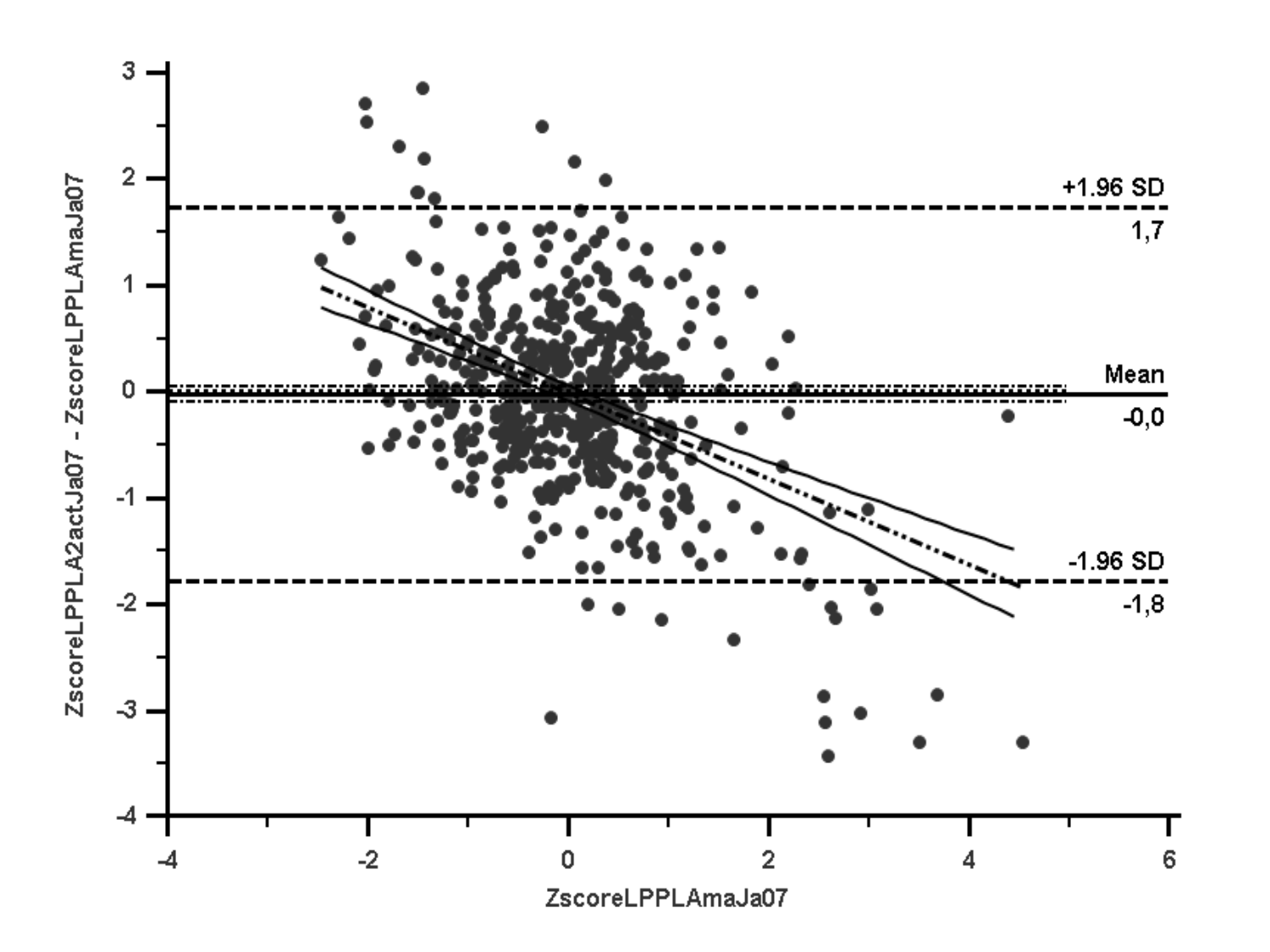

Supplement: Figure S1 — Lp-PLA2 Z-scores Bland and Altman plot. Increasing Lp-PLA2 mass z-score is associated with more negative values of the difference between Lp-PLA2 activity and mass and vice versa. The most extremes Lp-PLA2 mass Z-score values fell outside the confidence interval. Methods . We explored the relationship between lipoprotein-associated phospholipase A2 (Lp-PLA2) mass and activity by using Bland and Altman plot. Given the different units of measures of the two assays we computed their standardized scores (Z-score) in order to avoid generating an artificial proportional error. The z-score of the Lp-PLA2 mass vs. the difference of the Lp-PLA2 activity and mass z-scores was therefore plotted. Results. As shown in the supplemental figure, increasing levels of the Lp-PLA2 mass z-score corresponded to more negative values of the difference between Lp-PLA2 activity and mass and vice versa. Moreover, the most extremes Lp-PLA2 mass Z-score values fell outside the confidence interval. These results indicate that the proportion of the active counterpart of Lp-PLA2 varies much less than that of Lp-PLA2 mass and therefore that he biological effects of the enzyme are only partially accounted for by its circulating mass. These findings might explain why plasma Lp-PLA2 activity and not mass had prognostic value on cardiovascular events. (PPTX) [file pone.0048171.s001.pptx]
