# Supplementary material for: Lipoprotein-Associated Phospholipase A2 Activity Predicts Cardiovascular Events in High Risk Coronary Artery Disease Patients
Source: PLoS One. 2012 Oct 31;7(10):e48171. doi: 10.1371/journal.pone.0048171 (PMC3485195; doi:10.1371/journal.pone.0048171)
Supplement: Table S1 — Standardized differences of low and high Lp-PLA2 activity after matching. (DOC) [file pone.0048171.s002.doc]

**Supplemental Table S1. Standardized differences of low and high Lp-PLA2 activity after matching.** BMI: body mass index; HDL: high density lipoprotein; LDL: low density lipoprotein; EF: ejection fraction; CAD: coronary artery disease; AMI: acute myocardial infarction; ACE: angiotensin converting enzyme; ARB: angiotensin II receptor blocker.

| **Variable** | **Low Lp-PLA2 activity (n=126)** | **High Lp-PLA2 activity (n=126)** | **Standardized Differences** |
| --- | --- | --- | --- |
| Age (yrs) | 62.8±10.2 | 63.5±10.5 | 0.08 |
| Gender M/F (%) | 91/9 | 86/14 | 0.15 |
| BMI (Kg/m2) | 27.2±3.6 | 27.4±4.0 | 0.02 |
| S Creatinine (micromol/L) | 96.8±27.1 | 95.9±29.0 | 0.04 |
| HDL-Cholesterol, (mg/dL) | 43±9 | 44±10 | 0.05 |
| LDL Cholesterol, (mg/dL) | 141±33 | 143±36 | 0.03 |
| Triglycerides, (mg/dL) | 158±87 | 152±73 | 0.02 |
| Homocysteine, (µmol/L) | 13.6±7 | 14.3±9 | 0.008 |
| Left Ventricular EF (%) | 60±16 | 62±15 | 0.11 |
| Duke CAD score | 38±18 | 37±21 | 0.04 |
| Follow-up (years) | 7.0±2.5 | 7.2±2.4 | 0.08 |
| History of diabetes mellitus (%) | 12 | 13 | 0.02 |
| History of smoking (%) | 69 | 62 | 0.13 |
| History of hypertension (%) | 62 | 62 | 0 |
| History of AMI (%) | 36 | 37 | 0.01 |
| Aspirin therapy (%) | 80 | 80 | 0 |
| Beta blockers therapy (%) | 41 | 40 | 0.03 |
| ACE inhibitors therapy (%) | 53 | 53 | 0 |
| ARB therapy (%) | 2 | 3 | 0.10 |
| Heparin therapy (%) | 17 | 21 | 0.10 |
| Statins therapy (%) | 23 | 27 | 0.09 |
| Fibrates therapy (%) | 3 | 2 | 0.05 |
