# Supplementary material for: Lipoprotein-Associated Phospholipase A2 Activity Predicts Cardiovascular Events in High Risk Coronary Artery Disease Patients
Source: PLoS One. 2012 Oct 31;7(10):e48171. doi: 10.1371/journal.pone.0048171 (PMC3485195; doi:10.1371/journal.pone.0048171)
Supplement: Data S1 — Lp-PLA2 mass and activity Cox's regression analyses. (DOC) [file pone.0048171.s003.doc]

**Methods**

The predictors of cardiovascular mortality were investigated with Cox stepwise (backward, Wald) regression analysis, using inclusion and exclusion criteria of 0.05 and 0.10, respectively. The variables were entered in blocks and at each one the covariates meeting the exclusion criteria were removed before proceeding with the next block. The blocks were designed to avoid the inclusion of more than six variables simultaneously, because the number of deaths observed did not permit the inclusion of more than six variables in each model. The following blocks were used: first block included age and BMI; the second block LDL- and HDL-cholesterol, estimated glomerular filtration rate; the third block statin therapy; the fourth block left ventricular ejection fraction and Duke Prognostic Index score; the last block Lp-PLA2 either mass or activity.

**Results**

At Cox regression analysis Lp-PLA2 activity was a predictor of cardiovascular death (OR 1.011, 95% CI 1.001-1.021, p = 0.025) along with age (OR 1.069, 95% CI 1.035-1.103, p < 0.0001), left ventricular ejection fraction (OR 0.957, 95% CI 0.941-0.972, p < 0.0001), CAD Duke index score (OR 1.015, 95% CI 1.001-1.029, p = 0.036). Moreover Lp-PLA2 activity predicted acute myocardial infarction (OR 1.013, 95% CI 1.003-1.023, p = 0.009) along with left ventricular ejection fraction (OR 0.963, 95% CI 0.947-0.980, p < 0.0001). However, it was a predictor neither of cardiovascular events (OR 1.005, 95% CI 0.999-1.012, p = NS) nor of acute coronary syndrome (OR 1.006, 95% CI 0.999-1.014, p = NS). Lp-PLA2 mass was not a predictor of any of the aforementioned events at Cox regression analysis.
